# Toward a Unified Sub-symbolic Computational Theory of Cognition

**DOI:** 10.3389/fpsyg.2016.00925

**Published:** 2016-06-21

**Authors:** Martin V. Butz

**Affiliations:** Cognitive Modeling, Department of Computer Science and Department of Psychology, Eberhard Karls University of TübingenTübingen, Germany

**Keywords:** embodiment, predictive coding, free energy-based inference, anticipatory behavior, planning, learning, homeostasis, conceptualization

## Abstract

This paper proposes how various disciplinary theories of cognition may be combined into a unifying, sub-symbolic, computational theory of cognition. The following theories are considered for integration: psychological theories, including the theory of event coding, event segmentation theory, the theory of anticipatory behavioral control, and concept development; artificial intelligence and machine learning theories, including reinforcement learning and generative artificial neural networks; and theories from theoretical and computational neuroscience, including predictive coding and free energy-based inference. In the light of such a potential unification, it is discussed how abstract cognitive, conceptualized knowledge and understanding may be learned from actively gathered sensorimotor experiences. The unification rests on the free energy-based inference principle, which essentially implies that the brain builds a predictive, generative model of its environment. Neural activity-oriented inference causes the continuous adaptation of the currently active predictive encodings. Neural structure-oriented inference causes the longer term adaptation of the developing generative model as a whole. Finally, active inference strives for maintaining internal homeostasis, causing goal-directed motor behavior. To learn abstract, hierarchical encodings, however, it is proposed that free energy-based inference needs to be enhanced with structural priors, which bias cognitive development toward the formation of particular, behaviorally suitable encoding structures. As a result, it is hypothesized how abstract concepts can develop from, and thus how they are structured by and grounded in, sensorimotor experiences. Moreover, it is sketched-out how symbol-like thought can be generated by a temporarily active set of predictive encodings, which constitute a distributed neural attractor in the form of an interactive free-energy minimum. The activated, interactive network attractor essentially characterizes the semantics of a concept or a concept composition, such as an actual or imagined situation in our environment. Temporal successions of attractors then encode unfolding semantics, which may be generated by a behavioral or mental interaction with an actual or imagined situation in our environment. Implications, further predictions, possible verification, and falsifications, as well as potential enhancements into a fully spelled-out unified theory of cognition are discussed at the end of the paper.

## 1. Introduction

Theories on *embodied cognition* (EC) have come a long way (Lakoff and Johnson, 1980, 1999; Barsalou, 1999; Bergen, 2012; Clark, 2013). In their simplest form, they are perceived as the fact that cognition is influenced by the body. In more elaborate treatises, EC is typically differentiated into (a) *embodiment* itself, which focuses on how the body with its particular sensory and motor capabilities and its physical properties shapes the way we think, (b) *grounded cognition*, which emphasizes that our experiences are grounded in our physical world with its particular properties, and (c) *situatedness*, which points out that our experiences are also strongly influenced by our culture, society, and language (Pezzulo et al., 2013). Barsalou's simulation hypothesis (Barsalou, 1999, 2008) characterizes embodied cognitive states as *situated simulations*, which temporarily activate—or *re-enact*—particular situations, entities, or events by means of a corresponding set of embodied, modal neural activities. When we think about or actually perceive a certain situation, we simulate the crucial properties of this situation in our brain by constructing an approximate *mental image*. In very concrete situations—such as, for example, “thumbs up”—the imagining not only involves the associated, culturally conventionalized positivity and confirming implications, but also our own motor system, which simulates the thumbs pointing upwards, as well as a somewhat abstracted visual image of the gesture—to individually differing extents and vividness. However, in much more abstract situations, such as “a democracy,” there is still significant doubt if and to what extent EC contributes to the understanding of such concepts (Arbib et al., 2014).

As also pointed out by Arbib et al. (2014), Pezzulo et al. (2013), and others (cf. e.g., Bergen, 2012; Clark, 2013), a big problem with current theories of EC is that the focus mainly lies on where and to what extent indications for EC can be uncovered, typically attempting to explain the findings in a qualitative fashion. Actual quantitative cognitive theories and confirmations of such theories by means of cognitive system implementations are still largely missing. Thus, quantitative theories—or even better, neuro-cognitive models—of embodiment are needed to shed more concrete light on EC and its implications for cognition as a whole.

To develop such a computational theory, I propose to integrate the insights gained from EC into the theoretical frameworks of predictive coding (Rao and Ballard, 1998; Friston, 2002; König and Krüger, 2006; Kilner et al., 2007), free energy-based inference (Friston, 2005, 2008, 2010; Bastos et al., 2012; Adams et al., 2013; Friston et al., 2015), anticipatory behavior (Hoffmann, 1993, 2003; Butz et al., 2003; Butz, 2008; Pezzulo et al., 2009; Engel et al., 2013), events and event segmentation (Hommel et al., 2001; Zacks et al., 2007), and cognitive development (Konczak et al., 1997; Mandler, 2004; von Hofsten, 2004; Rochat, 2010; Mandler, 2012). In particular, I submit that the principle of free energy-based inference, which generally subsumes predictive coding and anticipatory behavior, should be enhanced with suitable structural information processing biases and event segmentation biases. I propose that these biases will enable the systematic development of a conceptual understanding of our environment, which allows the generation of compositional, conceptual thoughts. By biasing the development toward the maintenance of internal homeostasis, active inference will furthermore bias behavioral exploration and thus learning toward developing behavior- and motivation-oriented conceptual structures. As a result of the structurally-biased free energy-based inference processes, the development of particular types of predictive encodings can be expected to be involved and to be selectively activated while interacting with or thinking about the environment.

### 1.1. Theory background

Theories on *predictive coding* have at their premise the assumption that top-down predictions constitute perceptions while bottom-up signals are akin to error signals that modulate top-down predictions. *Free energy-based inference* mechanisms were shown to not only lead to neural activity adaptation and neural learning, but also to active inference, which causes the generation of epistemic and goal-directed motor behavior (Friston et al., 2010, 2014, 2015).

*Anticipatory behavior control* theories are closely related to predictive encoding and active inference, but they more explicitly emphasize that behavior needs to be inherently goal-directed, striving to satisfy bodily and cognitive needs. Behavior is invoked highly flexibly dependent on both the system's current needs and associated goals and the considered environmental circumstances. Anticipatory behavior control theories furthermore emphasize that behavior is controlled by the currently desired sensory consequences. During behavioral control, the focus lies on causing the desired sensory consequences, not on the control of the motor activity itself (Prinz, 1990; Hoffmann, 1993). This focus on action consequences is believed to lead to *common codes*, which specify motor actions and their—possibly multimodal—sensory consequences (Hommel et al., 2001). For example, high motor strength is associated with high volume in the auditory modality, fast acceleration and motion in the visual modality, and high pressure in the tactile modality (Prinz, 1997; Elsner and Hommel, 2001; Kunde, 2001). These insights have also fostered the theory of event segmentation, which highlights that dynamically unfolding episodes are systematically segmented into events and event boundaries (Zacks and Tversky, 2001; Zacks et al., 2007).

Developmental psychologists have shown that infants right after birth show indications of a rudimentary, postural body image and of anticipations about the sensory consequences generated by self-motion (Rochat, 2010). Moreover, the behavior of an infant has been shown to be goal-directed from the first months onwards (Konczak, 2004; von Hofsten, 2004). Finally, fundamental conceptualizations are provably present in infants and young toddlers, and have thus sometimes been termed “innate” conceptual primitives (Mandler, 2004, 2012). The unification of several theories of cognition in this paper implies how it may be possible to learn such “innate” conceptual primitives very early in life, starting with a progressively accurate predictive knowledge about the functionality of one's own body.

### 1.2. Contributions

The main aim of this paper is to sketch out a potential unification of several disciplinary theories of cognition into one, unifying computational, sub-symbolic theory of cognition. As the starting point, Andy Clark's (Clark, 2013) and others' proposition is generally agreed upon, that is, predictive encodings and free energy-based minimization can lead to the development of embodied cognitive systems. In addition, though, the proposed unification emphasizes that further learning biases are needed, which can be generated by including structural priors in the unfolding neural activities and wiring adaptations. Initial predictive encodings are inevitably bodily grounded in different sensory and motor modalities and in different frames of reference. To build more elaborate predictive encodings within and across modalities, however, the unification suggests that distinct predictive structures need to be developed. Motivated by the different disciplinary theories, the unified theory proposes how and which different forms of encodings should develop as well as how neuro-cognitive processing can continuously unfold within these encodings, further shaping them. As a result, the unification proposes how humans may be capable of developing and generating conceptual thoughts and abstract forms of imaginations, as well as self-motivated, goal-directed behavior by means of a distributed, highly-interactive network of distinct, and selectively partially activated predictive encodings.

In contrast to previous, unifying theories of cognition, such as SOAR (Newell, 1990) or ACT-R (Anderson, 1993), the proposed unification grounds symbols and production rules in neural structures and unfolding neural dynamics. Essentially, the unification emphasizes that rule-like and symbol-like structures need to be and can be encoded by distributed neural attractors, which approximate free energy minima. Thus, the proposal does not contradict these previous theories, but it additionally suggests how and which symbolic and rule-oriented structures can be generated by sub-symbolic, neural encodings, which are learned from and thus grounded in the gathered sensorimotor experiences during cognitive development.

### 1.3. Roadmap

**Figure 1:**
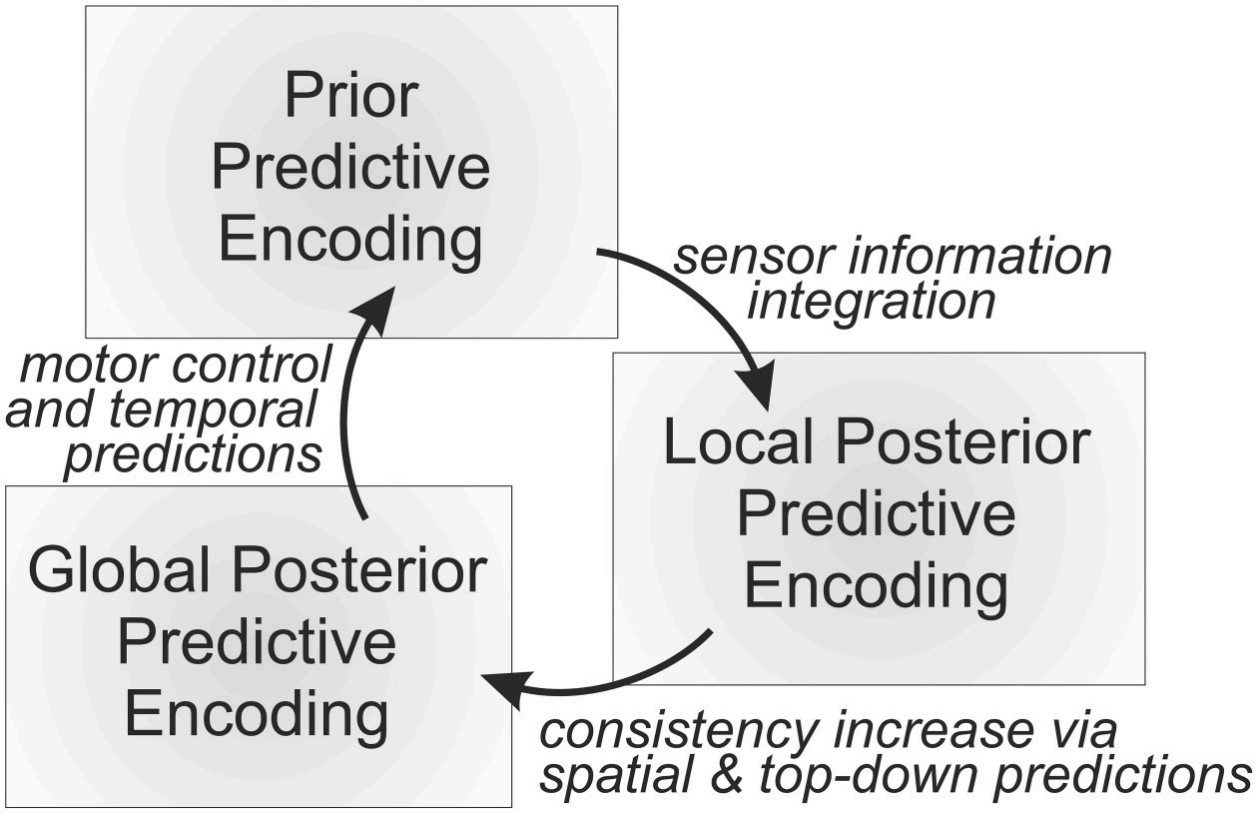
**An overall predictive processing loop continuously generates temporal predictions, compares the resulting distributed prior probabilistic state estimate with the incoming sensory information, and fuses the independent information sources yielding a distributed, probabilistic local posterior state estimation**. Finally, the internal active predictive encodings are adapted further toward establishing mutual consistencies, yielding an approximate global posterior distributed probabilistic state estimation. All the types of probabilistic state estimations are encoded sub-symbolically by means of neural activities, which essentially constitute the currently active predictive encodings.

**Figure 2:**
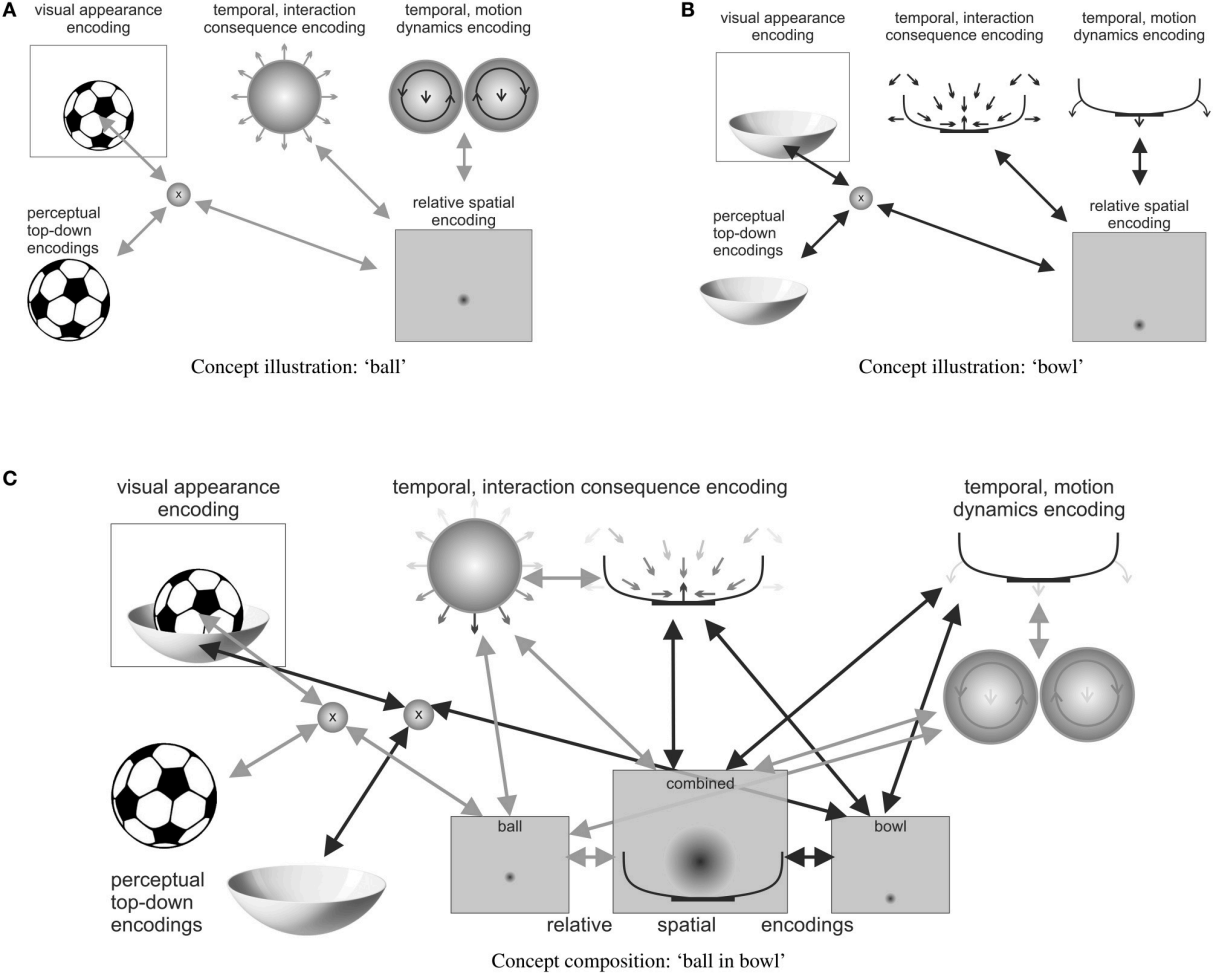
**A distributed illustration (highly simplified) of a possible predictive encoding of the compositional concept “a ball lies in a bowl,” including some of the most important active predictive encodings**. Note how the **(A)** ball and **(B)** bowl concepts are activated, sketching out the respective top-down predictions of their visual appearance as well as the respective temporal predictive encodings, which characterize potential motion interaction consequences of other items with the activated items. **(C)** The relative spatial predictive encodings together with the temporal predictive interaction encodings realize the “lies in” concept in the composition. While bidirectional arrows show ball- and bowl-respective active predictive encoding interactions, the darkness of arrows within individual illustrations indicates the current strength of activation. Note how, for example, the temporal interaction consequence encodings of ball and bowl approximately cancel each other out, thus generating a somewhat stable free energy minimum.

The remainder of this paper is structured as follows. First, it is sketched-out qualitatively how progressively abstract types of predictive encodings may develop from the gathered sensorimotor experiences. Second, it is explained how such predictive encodings may be learned by means of mathematical formalizations of free energy-based inference. Third, the focus lies on how goals and goal-directed behavior as well as attention and thoughts themselves can unfold by continuously and dynamically adapting the current set of active predictive encodings by means of an active, inference-based cognitive processing loop (cf. Figure 1). Fourth, examples of particular concepts and concept compositions illustrate the theory's prediction about how our brains think about a particular object or an object composition, such as “a ball lies in a bowl” (cf. Figure 2). Finally, the main propositions and predictions are summarized, including possibilities to further verify or falsify particular components. Moreover, it is discussed how social and language aspects may be incorporated and how actual implementations of the unifying theory of cognition may be accomplished.

## 2. Progressively developing particular predictive encodings

As is the custom on the psychological side, theories tend to be descriptive, and qualitative rather than quantitative or computational. In accordance to such approaches, this section proposes the unification of psychological, cognitive science, and neuroscience theories on a descriptive, qualitative level. Subsequent sections then sketch-out how the proposed and distinct predictive encodings may be learned, may develop, and may continuously unfold on a quantitative, computational level.

While formalizations of predictive encodings and free energy-based inference provide a general learning framework, they hardly distinguish particular types of predictive encodings. To foster suitable cognitive development, psychological theories have distinguished or selectively focused on (implicitly or explicitly) *temporal predictive encodings, spatial predictive encodings*, and *top-down predictive encodings* (Hoffmann, 1986, 1993; Prinz, 1990; Hommel et al., 2001; Zacks and Tversky, 2001; Knauff, 2013; Koffka, 2013). These may be considered the three fundamental types of predictive encodings, from which any more complex encoding can be constructed. By fostering the development of particular abstractions over these encodings, *event* and *event boundary encodings, event schemata*, and *episode encodings* may develop, which enable the formation of particular types of concepts. Besides the prediction that our brain develops these encodings, the theory unification also suggests that particular (genetically encoded) structural learning biases need be involved to ensure proper cognitive development. To be as precise as possible, the glossary in Table 1 may be consulted to clarify the conceptual meaning of the terminology used in this paper. The following sections provide details on how these types of encodings may develop from sensorimotor experiences.

**Table 1:** **Glossary of terminology used in the paper**.

Abstraction	An encoding that generalizes away from particular features in space and/or in time and/or over feature-specific aspects; typically, an abstracted encoding corresponds to a higher level, top-down predictive encoding
Active predictive encoding	An encoding that is currently active and that thus predicts the activity of other predictive encodings—just like a set of firing neurons that activate other neurons via their axons, the reached synapses, and the connected dendrites.
Cause	A physical property of an item, which may cause sensory signals and determine physical interactions (in analogy to Friston, 2010)
Concept	A subset of predictive encodings that specify (possibly relative) item properties, orientations, positions, and/or forces that are essential for a particular event to take place
Concept composition	A non-contradictory combination of concepts
Event schemata	An encoding of an event together with event boundary encodings that specify when the event can occur and how it typically ends (in analogy to Hard et al., 2006)
Dynamic event	An active set of temporal predictive encodings, which predict changes of causes, positions, and/or orientations of items in the environment, typically together with the forces that cause the changes, over an extended period of time
Episode encodings	A set of events and their typical ordering in time
Event	An active set of predictive encodings, which apply over an extended period of time (in analogy to Zacks and Tversky, 2001)
Event boundary	A particular state in the environment upon which one or several predictive encodings become applicable and/or one or several other predictive encodings are no longer applicable (in analogy to Zacks and Tversky, 2001)
Force	A physical force in the environment—including but not limited to motor activity—which causes items to change
Item	A body, body-part, object, material, thing, sensor, muscle, etc., that is, anything that exists in the environment and that can interact with other items
Modality	Sensory or motor signals provided by the respective sensors or activators
Module	A set of predictive encodings that integrates particular sensory and/or motor encodings or abstractions of such encodings in a particular manner
Orientation	Angular information about an item in the environment relative to other items in the environment
Position	Localization of an item in the environment relative to other items in the environment
Predictive encoding	Any form of predictive, neural encoding, which—when active—predicts the activity of other encodings—akin to a neuron or a set of neurons including the connectivity to other neurons via axon, synapses, and connected dendrites
Spatial predictive encodings	Predictions that map other predictive encodings onto each other
Static event	An active set of spatial and top-down predictive encodings of causes, positions, and/or orientations of items in the environment over an extended period of time
Temporal predictive encodings	Predictions forward in time, that is, predictions about changes in causes, positions, and/or orientations of items in the environment due to forces
Top-down predictive encodings	Predictions about more sensory- or motor-grounded signals in more abstract, generalizing forms, typically involving sensory/feature abstractions

### 2.1. From sensorimotor to general temporal predictive encodings

Starting from an embryonic stage, an important challenge for cognitive development inside the womb lies in learning as much about one's own body and its boundaries as possible. As Rochat (2010) points out, at birth, infants know, for example, when their thumb reaches their mouth—opening their mouth in anticipation of thumb insertion before the thumb actually touches the lips. Similarly, the rooting' reflex lets infants orient their mouth toward a touch on the cheek, but not when their own fingers touch it. Both observations indicate that a postural body image is at play, which perceives the touch as a self-touch—thus inhibiting the reflex or rather the further processing of the touch stimulus—and that the thumb's location relative to the mouth is (i) processed in an anticipatory manner and (ii) controlled in a goal-oriented manner.

Various researchers have investigated properties of a postural body schema and its plasticity (Holmes and Spence, 2004; Cardinali et al., 2009; Hoffmann et al., 2010; Butz et al., 2010b, 2014)^1^. What these treatises all have in common is that temporal predictive sensorimotor structures are learned, which predict sensory changes given motor activities or other sensory dynamics. Moreover, posture-dependent spatial mappings are learned, which enable the versatile projection of sensory stimuli into other frames of references and other sensory and motor modalities.

The proposition of learning predictive sensorimotor structures to be able to invoke goal-directed behavior dates back at least to the 19th century and has become known as the ideomotor principle (Herbart, 1825; James, 1890; Stock and Stock, 2004). The main proposition is that initially, purely reflex-like actions are paired with their sensory effects. Later, when the effects become desirable, the structure is assumed to enable the invocation of the motor activity that has previously produced the now desired effect. From the related perspective of forward-inverse sensorimotor models (Jordan and Rumelhart, 1992; Wolpert and Kawato, 1998; Haruno et al., 2001), forward-inverse model pairs are learned in order to be able to flexibly invoke inverse motor control to generate particular forward dynamics, and possibly to additionally apply appropriate sensor fusion (Schilling and Cruse, 2012; Ehrenfeld and Butz, 2013). Due to the problem of inverting forward kinematics and resolving redundancies on the fly, various forms of representation and redundancy resolution mechanisms have been proposed (Cisek, 2006; Butz et al., 2007; Stalph and Butz, 2012). Thus, sensorimotor-based temporal predictive models play at least a dual role: first, they are useful to filter behavior-induced sensory consequences according to the reafference principle in psychology (von Holst and Mittelstaedt, 1950); second, by inverting the temporal predictions, goal-oriented behavior can be induced by striving for those sensory changes that are expected to lead toward a desired goal state.

As motor activities essentially create forces in the environment, sensorimotor predictions can be generalized to *sensoriforce* predictions, which predict which perceivable changes in the environment can be generated by which particular forces. On the other hand, when abstracting away from concrete sensory changes, longer term, conceptualized changes are predicted. For example, the act of “pouring something into a container” abstracts away from the concrete substance (e.g., water, milk, or sand), from the concrete type of container (e.g., glass, mug, or bucket), and from the concrete motor actions that accomplishes the pouring (e.g., hand, both arms and hands, or machine).

Starting with sensor and motor encodings, *temporal predictive encodings* may develop, which predict how particular types of forces can lead to particular types of changes in the environment. Sensorimotor predictive encodings are thus the simplest form of temporal predictive encodings and support the development of more abstract temporal predictive encodings. Temporal predictive encodings may develop essentially anywhere where inferable forces change causes, positions, or orientations of items in the environment. After some learning, the mere observation of a particular control process or of a particular force can then lead to the invocation of accurate predictions of item changes. Temporal predictive encodings are closely related to the common codes proposed by the theory of event coding (Hommel et al., 2001). In the following, I contrast temporal predictive encodings with two other types of fundamental predictive encodings.

### 2.2. Three fundamental types of predictive encodings

When noting how the body senses the environment in different sensory modalities and over time, the following very fundamental and rather easily perceivable world properties foster the development of particular predictive encodings. These include predictions about relative sensory-grounded perceptions and their multimodal correlations, i.e., spatial predictive encodings, predictions about particular features and relative feature constellations, i.e., top-down predictive encodings, and changes in the activity of one or both of them over time, i.e., temporal predictive encodings.

*Spatial predictive encodings* can be bootstrapped by enforcing the learning of structural mappings between different sensory modalities. Since the various modalities are grounded in various, sensor-specific frames of reference (e.g., skin surface, retina, etc.), sensory causes, which are due to the presence of particular items in the environment, and which are often perceived in multiple modalities concurrently, may be correlated with each other to enable sensor fusion. Additionally, as these mappings depend on the current posture of the body as well as on its position relative to the outside environment, the currently active spatial predictive encodings must depend on current body posture estimates. Thus, generally speaking, spatial predictive encodings specify spatial relations that allow the mapping of different frames of reference onto each other body posture dependently.

For example, a keyboard may be co-perceived in the form of a retinotopic image by the eyes as well as in the form of tactile signals perceived by the typing fingers, both providing information about the current key positions relative to the body mid-axis. While the information from the eyes needs to be translated respective to the current eye fixation as well as the posture of the head relative to the trunk, the information from the fingers needs to be translated respective to the current finger, hand, arm, and shoulder postures. Both sources of information—from the eyes and fingers—are continuously mapped onto each other, leading to surprise signals given visual or tactile sensory feedback that significantly violates the expectations.

Various multimodal inference studies have shown (Maravita et al., 2003; Butz et al., 2010b; Brozzoli et al., 2014) that visual and tactile information interacts in various, body-centered frames of reference. The involved spatial predictive encodings appear to be found mainly in posterior parietal brain regions, which may be in this sense characterized as (but not restricted to) being the hub where different frames of reference are matched with each other and where different items are put into relative frames of reference (Maravita et al., 2003; Holmes and Spence, 2004; Chafee et al., 2007; Schindler and Bartels, 2013). Note that such spatial predictive encodings may be recruited by other cognitive processes, such as spatial reasoning processes (Knauff, 2013), number cognition processes (Wood et al., 2008), or general representations of magnitude (Walsh, 2003).

*Top-down predictive encodings*, on the other hand, generalize over space and focus on feature constellations and thus on characteristic, higher-level perceptions. The encodings essentially form perceptual templates (like a Gestalt) (Koffka, 2013), which predict item-specific sensory signals. Common sensory perceptions, such as faces or particular objects, indeed appear to be bundled in the brain in the inferior temporal cortex and the fusiform face area. In these areas neurons have been identified that respond to particular items and identities, largely independent of both their current position and orientation in space, and of the concrete form of presentation (e.g., sketch, photograph, or name of person) (Quiroga et al., 2005). Thus, it appears plausible that these regions encode compressed item templates, which predict corresponding sensory signals or—particularly on higher levels—feature constellations, when the item is perceived with particular sensory modalities.

Thus, top-down predictive encodings anticipate sensory information or abstractions thereof. In a particular situation, the currently active top-down predictive encodings expect particular feature and sensory perceptions. In conjunction with currently active spatial predictive encodings, these expectations can be mapped onto relevant sensory modalities. Indeed, research results again indicate that parietal regions encode such mappings, where current neural activity estimate the current position and orientation of items relative to the own body (Glover, 2004; Schindler and Bartels, 2013).

*Temporal predictive encodings* then encode changes in items as well as in their position and orientation over time, starting from very immediate sensorimotor encodings as discussed above. For example, *item-specific causes*, such as an item's size, its weight, color, contents, shape, etc., can change due to particular *forces*, which act upon the item. Similarly, position and orientation of an item can change. Thus, in these cases temporal predictive *sensoriforce encodings* are expected to be formed, which identify the particular types of forces that typically lead to particular types of perceivable item changes. Note once more that sensoriforce encodings are more general than sensorimotor encodings: motor activities result in specific forces; but other items in the environment can also generate forces; temporal predictive encodings predict how items change given forces, regardless if these forces are self-generated by one's own motor system or are generated by other entities in the environment. Temporal predictive encodings thus enable the development of abstractions over sensory changes due to motor activities toward feature changes due to active forces, respectively. Below, even further abstractions are detailed.

Note that the two types of fundamental possible changes of items in the world are reflected by the dorsal and ventral perceptual processing pathways. The dorsal stream toward the posterior parietal cortex processes mainly spatial properties, presumably mainly for preparing appropriate interactions (Goodale and Milner, 1992). The ventral stream toward the posterior, inferior temporal cortex processes primarily object properties and identities (Mishkin et al., 1983; Goodale and Milner, 1992; Dijkerman and de Haan, 2007; Milner and Goodale, 2008).

### 2.3. Event segmentation

While a particular interaction with the environment unfolds, typically interaction-characteristic sensorimotor dynamics are experienced. Accordingly, the theory of event coding (Hommel et al., 2001) defines events as common codes of actions and the typically resulting sensory or abstracted, perceptual changes. The event codes are thus closely related to the temporal predictive encodings specified above. The theory of event codes, however, does not specify when an event starts and when it ends. This aspects was emphasized in the event segmentation theory (Zacks et al., 2007), which characterizes an event as “a segment of time at a given location that is conceived by an observer to have a beginning and an end” (Zacks and Tversky, 2001, p. 17). A unification of the two theories may be possible by generalizing the concept of an even code further by defining it as *a set of active predictive encodings*. The beginning and end of an event code are then marked by the activation or deactivation of (a significant part of) this particular set. Due to this set-based definition, particular types of events can be characterized by the particular types of predictive encodings that are included in the set. *Static events* are those where a consistent, non-empty set of spatial and top-down predictive encodings is active. *Dynamic events* are those where, in addition, a non-empty set of temporal predictive encodings is active, which predict changes in other predictive encodings of the event-specific set of encodings.

*Event boundaries* then, marking the beginning or the end of an event, may be characterized by event transitions, that is, fundamental, significant, lasting changes in the set of active predictive encodings. Movement onsets and offsets as well as sudden directional changes are well-characterizable in this manner. Moreover, temporary states without motion, such as when changing direction from forward to backwards, play a significant role during segmentation. For example, an object may disappear and then reappear (onset of top-down, object-specific spatial predictions), or it may move away but then turn around and thus move toward the observer (offset followed by onset of temporal predictive motion encodings). Similarly, a bottle may become light when emptied and become heavier when filled (changes in top-down predictive property encodings), or a walking person may suddenly start to run (changes in temporal predictive encodings).

With these definitions in hand, observations over time can be segmented into *events*, during which particular predictive encodings apply, and *event boundaries* or *event transitions*, which are marked by particular, significant changes in the set of active predictive encodings. Note how segmentations thus are able to detect a huge range of event boundaries, including motor activity of force onsets and offsets, behaviorally relevant changes in orientation or position (e.g., particular orientations may allow particular manipulations, thus leading to the onset of particular other predictive encodings), the appearance or disappearance of particular items, and even relevant changes in item properties (e.g., a bottle becomes empty, a bottle is opened etc.).

Interestingly, similar approaches to segmentation have been used in the robotics community to develop behavior-grounded language grammars (Pastra and Aloimonos, 2012; Dominey, 2013; Schilling and Narayanan, 2013) as well as to cluster types of object interactions into equivalence classes of the relative object changes that are encountered (Aksoy et al., 2011; Wörgötter et al., 2013). Similar action-grounded grammars have also been successfully used to create seemingly alive, knowledgeable, learning and behaving virtual agents (Ehrenfeld et al., 2015). The segmentation of predictive encodings into events and event boundaries thus seems to enable the development of embodied, grammatical, conceptual encodings of the environment.

### 2.4. Event schemata

Segmenting predictive encodings over time into events and event boundaries will develop encodings that specify the conditions necessary to start an event, the final results at the end of an event, and the predictive encodings that are active while the event unfolds. When combining condition, event, and final result encodings, *event schema* encodings can develop. Related ideas have been put forward in relation to the theory of event segmentation, developing event schemata (Hard et al., 2006; Zacks and Tversky, 2001; Zacks, 2004; Zacks et al., 2007), in relation to the theory of anticipatory behavioral control, which specifies how condition-action-effect schemata can be learned (Hoffmann, 1993; Hoffmann et al., 2007), and in relation to general representations of knowledge and reasoning (Rumelhart and Ortony, 1977; Barsalou, 1999). In terms of predictive encodings, event schemata can be specified as the following triple of predictive encoding clusters:

1. *conditional predictive encodings*, which identify those spatial, top-down, and temporal predictive encodings that are necessary to allow the activation of an event;
2. *event encodings*, which characterize the particular unfolding event including the involved forces and item changes;
3. *final event encodings*, which specify the predictive encodings that signal the end of the unfolding event.

As a result, an event schema systematically encodes under which circumstances an event can take place, the characteristics of the event itself, and under which circumstances the event will stop to unfold.

The following example of two successive event schemata may help to clarify the structure of these encodings: Consider reaching for an object to establish contact. The conditional encoding must signal that an object is present and in a reachable distance, that is, the predictive spatial encoding temporarily associated with the object must signal that the relative distance to the body is shorter than one's own arm length. Also, one of the arms must be available to execute the reaching motion. The dynamic event then characterizes the motion of the arm needed to approach the object as well as the force needed to exert this motion, that is, the forces that minimize the relative spatial distance between hand and object. Note that the force encodings may be converted into actual motor commands when the action begins to unfold, considering the current state of the body, the concrete object position, etc. The final event encodes the establishment of touch as well as the signal that the distance between hand and object reaches zero.

Next, let us now consider the consequent touch event. The conditional encoding here corresponds to the just characterized event-boundary, encoding that a body part comes into contact with something else (possibly another body part) in the environment, that is, the relative distance reaches zero. The event itself in this case is characterized by the intensity and other properties of the touch as well as the involved forces, in the form of pressure encodings between a body part and another item. The final event encodings characterize the result of the touch, including possible immediate release and thus a relative distance to the object that increases, or the maintenance of tactile feedback, triggering another event schema such as “holding onto something,” or “stroking something.”

When particular events or event transitions are desired—say due to the internal motivational, homeostatic state of the agent (see later)—the proposed structure enables the activation of those predictive models that are known to initiate the desired event or that lead to the desired event transition. Due to the proposed structure of event schemata, it then becomes possible to chain such encodings inversely, striving to establish relevant conditional encodings in order to ultimately achieve the final event. For example, when no food is in reach but food consumption is the final goal, first food needs to be found and moved into reach. Note how this proposition is akin to hierarchical, model-based reinforcement learning architectures (Sutton et al., 1999; Konidaris et al., 2011; Botvinick and Weinstein, 2014). Similar encoding structures were also proposed by Zacks et al. (2007), who characterized them as highly suitable goal-directed planning and inference structures for deducing the current goals and intentions of observed others.

### 2.5. Abstraction and hierarchical structuring

Given event schemata, even higher-level, top-down predictive encodings may be developed. Frequently encountered types of interactions may be clustered into *episode encodings*, where the simplest kinds of such episodes may be characterized by particular bodily interactions with the world, including, for example, eating, drinking, scratching, walking, or grasping to hold. In all these cases, several event schemata can be clustered into one predictive code, which characterizes a particular interaction including how it typically unfolds over time in the form of a set of event schemata. It thus predicts the dynamic activity of a set of temporal, top-down, and spatial predictive components.

Eating, as perceived by an infant, for example, may start with suction in the appropriate situation, may unfold by continued suction, the perceived effect of milk flow into the mouth—or, more sensor-orientedly speaking, the sensation of a warm fluid substance inside the infant's mouth—and the motor act of swallowing, with the resulting changes of decreased fluid presence in the mouth, warm feeling inside the stomach, and the perception of rewarding signals sent to the brain by the stomach (cf. e.g., Butz, 2013). Finally, this unfolding may cease resulting in a mouth without milk, no more milk inflow, no more swallowing of milk, etc. Thus, a feeding episode was described in terms of event schemata, including the involved predictive encodings and their interactions over time. Due to the re-occurrence of such episodes—and most likely also due to the motivational and emotional significance of particular episodes—progressive further conceptualizations may be enabled, converging to compact encodings of those predictive encodings that need to be active to encounter particular interaction episodes. In this manner, for example, encodings of the hardness of an object may develop, co-determining, for example, its suitability to be used as a hammer for driving a nail into a wall.

Note how—once suitably compressed into episode encodings in this way—such episodes can be imagined when decoupled sufficiently from the current sensory perceptions. Moreover, one can strive to accomplish whole episode encodings in a goal-directed manner. Besides the possibility of pursuing interaction episodes, sufficient observations of other people executing seemingly similar interaction episodes can be comprehended by means of simulating the apparently corresponding interaction episode, continuously comparing it with the observations and filling in missing observations. Finally, interaction episodes may also be paired in parallel or in sequence with other episodes, or even recursively with themselves, enabling the formation of ever more abstracted encodings of interaction episodes, such as “attending a lecture” while “studying at the university” while “working on ones own career.”

Note also how distinct predictive encodings often co-occur systematically, and how such co-occurrences are thus suggestive about which type of more general, abstract event or episode currently unfolds. Thus, even when only observing some aspects of an event or a chain of events, inference processes are able to deduce—or at least are able to make an educated guess—about which actual episode is currently observed and thus about which goal is currently being pursued by the observed agent (Zacks and Tversky, 2001; Zacks et al., 2007). Examples are the observation of a pantomimed interaction, of a partially occluded interaction, or also of a short snapshot of an action, for example shown in a movie, that implies a complex episode spanning hours, days, or even years.

## 3. Learning by formulations of free energy-based inference

Free energy-based inference offers itself as the fundamental neural processing and adaptation principle (Friston, 2005, 2010; König and Krüger, 2006), subsuming principles such as the Bayesian brain (Knill and Pouget, 2004; Doya et al., 2007) and predictive encoding (Rao and Ballard, 1998), and allowing the derivation of state-of-the-art machine learning techniques (Friston, 2010), including neural activity adaptations and learning in neural networks. When desired future states are integrated into the free energy formulations, active inference mechanisms can be generated, which cause the execution of epistemic, information-gain oriented, curious behavior and goal-oriented behavior (Friston et al., 2010, 2015).

Loosely speaking, formulations of free energy-based inference suggest that the brain focuses on predicting incoming sensory information, thus canceling-out or “explaining away” the sensory information that was predicted. Only the error is propagated “upwards” through a supposed hierarchy of processing stages. Thus, while top-down predictions encode the actual sensory information, bottom-up information encodes the error, that is, the residual that is left after subtracting or dividing the top-down prediction from the bottom-up information. As a consequence, free-energy formulations yield *generative models*, which are capable of filling in absent sensory, motor, or abstracted information and which are thus generally capable of generating “imaginations” of particular items, interactions, and even whole situations and episodes.

Various other researchers have suggested that predictive encodings combined with free energy-based inference naturally offer themselves to give embodied theories of cognition a computational backbone, and this proposition has been widely discussed for several years now (Clark, 2013). While the proposition in this paper is thus not new, it focuses on the types of structures, their interactions, and abstractions that typically develop. In the following, I put predictive encodings and free energy-based inference mechanisms in the light of the developing predictive encodings discussed above, showing how they can be learned, and how neural dynamics and behavioral control can continuously unfold within them.

### 3.1. Learning different types of predictive encodings

To give the reader a feel of what predictive encodings are about mathematically, and how they are a consequences of free energy-based formulations, the following is a short mathematical introduction to predictive encodings. The goal is to show that the general formulation is relatively simple and can generate top-down, temporal, and spatial predictive encodings. Depending on precision estimates, predictions may even overrule bottom-up sensory evidence. Moreover, the formalization shows how resulting error signals can yield both, neural activity adaptation and structural, weight adaptation (fast and slow error-based adaptations, respectively).

Let us start with the most basic type of predictive encoding, that is, top-down predictive encodings, which develop from and generalize over bottom-up activation signals. This type of predictive encoding was first introduced as a neuro-vision architecture (Rao and Ballard, 1998). It can also be derived from more general free-energy based formalizations (Friston, 2002, 2010). Top-down predictive encodings can be formulated by starting from the most common one found in the original work of Rao and Ballard (1998), which was more recently unified with biased competition (Spratling, 2008, 2014). In this case, a strict hierarchy of layers *S*_*i*_ is assumed. Layer *S*_0_ =_*def*_*X* is the “lowest,” modal grounded, sensory input layer, which is fed with sensory signals *x*. The current neural activity in layer *S*_*i*_ is denoted by ${\text{y}}^{S_{i}}$ and connections from layer *i* − 1 to layer *i* are specified in the connection matrix ${\text{W}}^{S_{i}}$. Note how the current neural activity ${\text{y}}^{S_{i}}$ corresponds to a particular, currently active top-down predictive encoding, because the neural activity generates top-down predictions via the matrix ${\text{W}}^{S_{i}}$ in the next lower layer *S*_*i*−1_. Moreover, the weight matrices themselves determine which top-down predictive encodings can be actually generated (by a weighted combination of neural input activities), thus constituting all top-down predictive encodings that are possible. In sum, while the weight matrices determine the available predictive encodings and thus the overall predictive model of the system, the current neural activities determine the currently active predictive encodings.

Using this notation, the following update of the neural activities in a layer can be formulated:

(1) $$\begin{array}{r} {\text{y}}^{S_{i}}\leftarrow \left(1-\alpha -\beta \right){\text{y}}^{S_{i}}+\gamma {\text{W}}^{S_{i}}{\text{e}}^{S_{i-1}}+\beta {\left({\text{W}}^{S_{i+1}}\right)}^{T}{\text{y}}^{S_{i+1}}, \end{array}$$

where

(2) $$\begin{array}{r} {\text{e}}^{S_{i-1}}{=}_{\text{def}}{\text{y}}^{S_{i-1}}-{\left({\text{W}}^{S_{i}}\right)}^{T}{\text{y}}^{S_{i}}, \end{array}$$

specifies the error signal in a particular layer, defined as the difference between the top-down prediction and the currently active predictive encoding in a layer. In their original work, Rao and Ballard (1998) have shown that edge encodings similar to those found in *V*1 can develop when this update mechanism is paired with weight updates that strive to minimize the remaining errors ${\text{e}}^{S_{i}}$ in each layer (enforcing sparsity can further foster this development). Essentially, the resulting algorithm first applies several activity adaptations, which determine the currently active top-down predictive encodings while minimizing the error activities. Next, the algorithm adapts the weight values to minimize the residual error even further. In various later publications on learning in the area of vision as well as in other related learning tasks, Bayesian mechanisms were shown to approximate the predictive encoding approach and they were related to attentional modulation, multi-sensory integration, optimal decision making, and planning as probabilistic inference (Denève and Pouget, 2004; Rao, 2005; Körding and Wolpert, 2006; Doya et al., 2007; Botvinick and Toussaint, 2012).

Parameters α, β, and γ determine how the currently active encoding is combined with the top-down expectations and the bottom-up error signal. As Spratling (2008) has shown, a particular choice of parameters can generate system behavior that is akin to biased competition—disambiguating bottom-up information by means of top-down predictions (leading, for example, to the generation of imaginary contours in the Kanizsa Triangle illusion). Note, however, that the parameter values may also be changed adaptively, dependent on current precision estimates (i.e., inverse variances) and the agreement between the three signals (i.e., inverse estimation divergence). These observations essentially confirm that this formulation enables (i) the activity maintenance of predictive encodings during the lack of evidence by setting α = β = γ = 0, which should be the case when error information as well as top-down predictions are highly imprecise; (ii) the inclusion of top-down predictive influences including predictive coding-like updates by setting β > 0 as well as biased competition updates by setting β < 0, which should be the case when the top-down information is estimated to be less precise or more precise relative to the estimated precision in the currently active predictive encoding, respectively; (iii) bottom-up driven error corrections by increasing γ > 0, where larger bottom-up precision estimates should increase the bottom-up influence; (iv) the forgetting of current encodings by increasing α > 0, where an increase in α can be interpreted as an increase in encoding uncertainty.

These observations essentially show that predictive encodings, particularly when endowed with precision estimates, can generate top-down imaginations when the certainty, that is, the top-down precision estimates, are very high. Moreover, it shows how bottom-up error information generally interacts with top-down predictions dependent on the relative precision estimates. Free energy-based formalizations of these equations are not further spelled-out in this article, but formalizations are available, which also detail how precision estimates may be encoded by means of variational Bayesian approximations, exact Bayesian formalizations, or by neural population encodings (cf. e.g., Friston, 2002, 2010; Friston et al., 2010; Ehrenfeld et al., 2013; Kneissler et al., 2015). The following paragraphs detail how temporal and spatial predictive encodings can be realized by the same principle.

Besides the formulated strict hierarchy of top-down predictive encodings, temporal predictive encodings can be formulated in a similar manner (cf. e.g., Goodwin and Sin, 1984; Friston, 2008; Kneissler et al., 2015). Such formulations allow the derivation of extended versions of the Kalman filter and enable the simultaneous learning of temporal predictive encodings while optimally filtering state estimations (Kneissler et al., 2015). Memisevic (2013) has shown how to relate images using probabilistic temporal predictive encodings. In this case, multiplicative gates were used to flexibly wire input to output images, effectively generating a temporal predictive encoding for matching images. Abstractions over item interactions have been shown to lead to temporal predictive encodings that can characterize abstract object interactions, such as a push, a pull, or a grasp (Giese and Poggio, 2003; Fleischer et al., 2012).

Spatial predictive encodings across correlated sensory modalities have been developed with related approaches, where the different sensory modalities are grounded in particular, different frames of reference, thus posing the challenge to map frames-of-reference onto each other in a posture-dependent manner (Friston et al., 2010; Kneissler and Butz, 2014; Kneissler et al., 2014; Schrodt and Butz, 2015). Closely related purely visual artificial neural network models were show to be able to develop interactive spatial and top-down predictive encodings (Chikkerur et al., 2010; Bergmann and von der Malsburg, 2011; Memisevic, 2013; Fernandes and von der Malsburg, 2015).

In sum, formulations of predictive, generative encodings are available that enable the learning of top-down, spatial, and temporal predictive encodings. Combinations of such learning biases in a modularized fashion can foster the generation of hierarchical predictive encodings, spatial mappings of such encodings on abstract levels, as well the prediction of the changes in such encodings over various time scales. Closely related formulations of Restricted Boltzmann Machines (Smolensky, 1986), the development of fast learning algorithms for training them (Hinton et al., 2006), and enhancements enabling multiplicative interactions (Memisevic, 2013; Schrodt et al., 2015) suggest that state-of-the-art artificial neural network learning techniques can be employed to learn the described three fundamental types of predictive encodings.

### 3.2. Learning event-detectors via multiplicative, nonlinear gates

With the possibility of learning the three fundamental types of predictive encodings, the second challenge comes when events and event boundaries are to be detected. Various research directions have proposed solutions from an anticipatory behavior perspective (Fleischer et al., 2003; Butz et al., 2004; Herbort et al., 2005) as well as from a hierarchical reinforcement learning perspective (Simsek and Barto, 2004; Botvinick et al., 2009; Botvinick and Weinstein, 2014). In artificial neural networks, gating mechanisms have been developed that enable the extended sustenance of neurally encoded short-term memory items—the so-called long short-term memory (LSTM) networks (Hochreiter and Schmidhuber, 1997; Otte et al., 2015; Schmidhuber, 2015). LSTMs have been successfully applied to learn context-sensitive grammars (Hochreiter and Schmidhuber, 1997; Pérez-Ortiz et al., 2003) and even to solve speech recognition and automatic language translation tasks (Graves et al., 2013; Sutskever et al., 2014). Although at the moment LSTMs are always trained by means of backpropagation, in speech recognition and translation tasks they have been applied in a temporal and top-down generative manner—generating sequences of words that probabilistically appear to correspond to the auditory input or the word-wise input from another language. Thus, LSTM networks can be employed as predictive, generative models.

In LSTMs, nonlinear gates multiplicatively combine the activity of a linearly activated neuron with a strongly, non-linear activated one (e.g., sigmoidal). As a result, the input via the linear function tends to gather evidence while the non-linear input determines when the gathered information is passed on. Additionally, gathered information can be maintained via an identity-recurrence in LSTM *memory cells*, such that particular information can be actively maintained until further notice. LSTMs are thus highly suitable for developing event boundary detectors via nonlinear gates, while approximately linear encodings predict possible event progressions.

To foster the development of such event encodings further, an alternative or complementary approach is to incorporate explicit event boundary detectors. Event boundaries can be explicitly detected by monitoring the continuous activation of predictive encodings. When registering significant changes in the active encodings—for example, when activity ceases after an extended period of time of activation, or, vice versa, when activity commences and remains active after an extended period of near inactivity—then this signal can be interpreted as an event boundary signal. A similar approach has been used to detect doorways in the four-rooms hierarchical reinforcement learning problem, showing high detection robustness even with very large amounts of sensory noise (Butz et al., 2004).

Although the full derivation of such mechanisms by means of free energy-based inference principles remains as a future challenge, the available theory suggests that it is possible. Moreover, recent advancements in particular wiring manipulations differentiating “drivers” and “modulators,” combining them in a multiplicative fashion, have shown that interactions of the kinds described above can be realized by means of multiplicatively interacting predictive encodings (Spratling, 2014). The further targeted wiring of such neural architectures— especially when paired with the available computational power and large amounts of data, which may be gathered by simulated agents in virtual reality environments (Ehrenfeld et al., 2015; Mnih et al., 2015)—is bound to yield even more competent machines, which will be able to develop the event schemata and episode encodings detailed above.

## 4. Goal-directed behavior and cognition

While the previous section has sketched-out a path how distinct predictive encodings may be learned, the overall cognitive process that may cause these neural adaptations while interacting with the environment still needs to be specified. In this section, I show how, given the developing predictive encodings, inference-based planning, decision making, and control may be realized, including both, motor control and mental control (i.e., thinking). To do so, it is necessary to generate free energy internally, which can be accomplished by principles of homeostasis. Paired with active inference, thoughts and behavior can be generated, which are inherently directed toward maintaining body and mind in an approximately balanced, homeostatic state.

### 4.1. Active inference and homeostasis

It has recently been shown that free energy-based inference paired with encodings of future, desired states can generate *active inference* processes, which cause both, epistemic behavior as well as goal-directed behavior (Friston et al., 2014, 2015). Epistemic behavior essentially strives to minimize uncertainty about internal state estimations and the real state in the world, thus ensuring that desired states are reached with high certainty. Goal-directed behavior strives to minimize the difference—or divergence in terms of probability density encodings—between a desired, homeostatic state and the current state. As a result, active inference causes a system to act curiously in order to ensure behavioral success, while striving to maintain homeostasis.

In the behavioral psychology and neuroscience literature, it has been shown that humans exhibit approximately optimal decision making and behavior, in which uncertainties about the consequences of own behavior are taken into consideration (Trommershäuser et al., 2003; Cisek, 2006; Körding and Wolpert, 2006; Herbort et al., 2007). Theories of optimal control, which are closely related to active inference (Friston et al., 2010; Friston, 2011), can approximately model such behavior (Todorov, 2004). However, various cognitive science studies suggest that the brain only achieves approximate optimality at best. Our own recent research has shown that action decision making and control depends at least on prior knowledge about tools and objects, the position of the object relative to hand and body, the orientation of the object, further object properties, such as suitable grasp points, the initial, intermediate, and final goals of the interaction, as well as the position of obstacles and other items in the vicinity (Herbort and Butz, 2011; Herbort et al., 2014; Belardinelli et al., 2015, 2016a,b). Thus, behavioral decision making can be influenced by many factors. The research results also suggest that behavior is not fully optimized, or fully planned to the last detail, in each actual interaction; rather, heuristic habitual behavior is applied dependent on the task and the circumstances, and these habitual behaviors are adjusted when free, redundant degrees of freedom are available (Herbort and Butz, 2012, 2015). Along these lines, Cisek (2007) has put forward an *affordance competition hypothesis*, where objects are characterized as affording particular habitual interactions, which compete for activity resources dependent on current motivations and other priorities.

Note that such habitual interactions can be interpreted as motor primitives, somewhat similar to dynamic motion primitives that are used in the robotics community (Kober and Peters, 2011; Ijspeert et al., 2013). These motor primitives can be related to dynamic event encodings and can be integrated into event schema encodings, where a motor primitive is applicable given conditional encodings are satisfied. Comparisons of the achieved and desired final event can be used as the reinforcement learning signal. The proposed hierarchies of event schema and episode encodings offer themselves naturally for the activation of model-based, hierarchical reinforcement learning-based planning and decision making processes (Botvinick et al., 2009; Botvinick and Weinstein, 2014), which can be implemented by means of free energy-based active inference (Friston et al., 2010, 2014, 2015).

To generate self-motivated inference processes, internal homeostatic or Hullian motivational states (Hull, 1943) may drive the actual behavior (Hsiao and Roy, 2005; Konidaris and Barto, 2006; Butz et al., 2010a). Differences between the desired homeostatic state and the current state can be interpreted as free energy that asks for minimization, causing active inference. The more pressing the internal motivation is, that is, the larger the difference (or divergence in terms of probability densities) between the desired and the current homeostatic state, the higher is the free energy and thus the stronger the active inference processes, effectively “pulling” the system toward satisfying the internal motivation. Active inference consequently leads to the activation of those episode encodings, event schema encodings, the involved event encodings, and ultimately temporal predictive encodings of the system, which will lead to the generation of those forces and associated motor activities that are expected to satisfy the system's homeostatic states with high certainty.

### 4.2. Overall cognitive processing loop

While I have now clarified that active inference can lead to both, epistemic, information-seeking, curious behavior and goal-driven behavior, it remains to be shown how behavior may be selectively triggered given the current, predictively encoded situation of body and outside world. With the versatile behavioral capabilities we humans have, motivation-biased decisions need to be made with respect to the overall context. And indeed, as has been shown in Friston et al. (2015), the brain needs to make continuous decisions between epistemic actions for improving the accuracy or precision (inverse variance) of the current predictive encodings about the world, and goal-driven actions for satisfying motivational needs. To come to these decisions, the following overall neuro-cognitive processing loop fosters the self-maintenance of neural activities over time, continuously striving to maintain overall bodily homeostasis (including most likely neural-homeostasis).

The proposed neuro-cognitive processing loop is an extension of a particular Bayesian, predictive information processing architecture (Ehrenfeld and Butz, 2013; Ehrenfeld et al., 2013), called *modular modality frame* (MMF) architecture. MMF models the self-maintenance of an internal, concurrent and consistent, probabilistic postural and visual body image of an arm. It represents the arm state probabilistically by means of a set of Gaussians (Ehrenfeld and Butz, 2013) or by neural population codes (Ehrenfeld et al., 2013). To enable scalability, MMF represents the arm not in one frame of reference but in several, which are centered on individual arm limbs relative to other limbs. Consequently, each probabilistic, modularized state encoding covers only a two- or three-dimensional space, which ensures the scalability of MMF.

The overall MMF architecture then continuously integrates temporal forward predictions in the form of probabilistic approximations of local Jacobians to predict next body state estimation priors, typically yielding a slight decrease in estimation precision. Next, modal and modular (typically highly noisy) sensory information is provided, leading to information gain when fusing the prior state estimations with the incoming sensory information. During the process, the sensory information is compared across the spatial predictive models to be able to estimate current relative sensor information plausibility on the fly—a mechanism that may be applied in any layer including those without access to actual sensory information. The overall information fusion process then yields the local posterior estimate of the system, that is, a set of local posterior state estimations. Finally, these local posterior state estimations are compared pairwise across spatial predictive encodings, bringing the internal state estimates in further accordance with each other, depending on their relative precisions (inverse variances). Figure 1 shows this information processing loop.

Let us reconsider the described processing loop of MMF in light of the presented different types of predictive encodings. In this general case, the currently active temporal predictive encodings will be responsible for generating prior (next) active encodings on all available levels of abstractions. As in the MMF architecture, temporal predictions will typically result in a slight loss of precision, that is, in an increase in uncertainty (variance) in the consequently active predictive encodings. Next, bottom-up information will be fed into the system, leading to local information gain and a general upwards pass of prediction errors. During this process, information gain will be typically experienced and the system will adapt its active predictive encodings to better match the bottom-up, sensor-based information, thus yielding local posterior active predictive encodings.

Finally, the process needs to foster agreement between the currently active encodings to form an overall state estimate, which may be termed the *global posterior active predictive encoding*. That is, the predictive encoding activities are adjusted such that the global error that is generated by these encodings (the sum of all errors akin to Equation 3) is approximately minimized. As the predictive encoding system is essentially a specific kind of highly modular, distributed, restricted Boltzmann machine with additional processing and wiring biases, the global attractor is generally very hard to determine. Thus, the global attractor needs to be approximated by means of distributed but interactive local adjustments, which can be realized by mutually adapting predictive encoding activities given other, concurrently active, connected predictive encodings. The result is a system that strives to continuously activate those predictive encodings that are in maximal consistent agreement with the available sensory information as well as with the overall predictive encoding model.

### 4.3. An anticipatory, self-maintaining cognitive system

Coming back to behavior then, the global posterior encodings detailed in the cognitive processing loop above are the ones that can cause the execution of goal-directed behavior by means of active inference. Given an unbalanced homeostatic state, temporal predictive encodings will be activated (by active inference mechanisms), which predict a change in the homeostatic variables toward higher homeostasis. Thus, internal differences between current and desired homeostatic states “pull” the brain's neural activities toward generating more desired states and thus toward producing those motor activities that are believed to enable the causation of—or, when possible, to directly cause—the desired changes.

Behavior is thus embedded into a system that strives for the maintenance of homeostasis, akin to autopoietic systems proposed by Maturana and Varela (1980), but that has developed particular, predictive structures to be able to actively maintain homeostasis. The system is also a fully anticipatory system, in which the main anticipatory drive (Butz, 2008) comes from internal, mostly bodily grounded motivations (see e.g., Butz, 2013, for an overview). Behavior is thus viewed as actively unfolding temporal predictions that translate desired state changes into motor behavior.

Note that when abstracting from behavior to forces, behavioral execution is not necessary anymore such that processes of behavioral control may turn into processes of attentional control (Balkenius and Johansson, 2007; Balkenius et al., 2008). Allowing attentional control to predictively manipulate internal, abstract, predictive encodings paired with the maintenance of sufficiently large, local, distributed, predictive agreement (akin to a distributed, local minimum in free energy), these processes may lead to actually imagining particular unfolding forces even though these are currently not perceivable (cf. e.g., Schrodt and Butz, 2016). The self maintenance-oriented processes, paired with locally distributed, inference-based attention, can thus generate not only motor behavior but also purely internal activities, which constitute abstract thought processes.

### 4.4. Imaginations, concepts, and concept compositions

The unifying theory thus proposes a system that not only directs its motor activities but also its internal attention—and thus self-generated thoughts themselves—toward the maintenance of internal homeostasis. When sufficiently detached from current sensory perceptions and motor activities—which can be realized by decreasing the bottom-up error influence (parameter γ → 0 in Equation 1) and/or by inducing strong biased competition (by a large negative parameter β, cf. Equation 1)—the system may thus be able to imagine both, static events, scenes, and situations as well as dynamically unfolding events, collections of events, and episodes. These imaginations come in the form of distributed attractors, which are striven for by minimizing free energy between the involved, currently active predictive encodings.

Coordinated predictive information exchanges that minimize the free energy between the involved interactive predictive encodings may be realized in the brain by synchronizing the interacting firing patterns in various, distinct frequency bands, which are able to distinctively influence top-down and bottom-up information flow (Bastos et al., 2015; Fries, 2015). By means of the cognitive processing loop combined with attention on the currently active predictive encodings and on the homeostatically-activated goals, the system will explore the predictions of its activated predictive encodings in a self-motivated manner, altering them while striving for the maintenance of approximate free energy minima. If this is correct, activity changes in spatial predictive encodings would lead to considerations of alternative arrangements and relative perspectives. Similarly, activity changes in top-down predictive encodings would lead to considerations of involvements of other items and other causes. When processing temporal predictive encodings, the consequences of particular environmental force-driven interactions would be considered in the imagined situation. As a result, not only behavior but also abstract thoughts—including planning, perspective taking, memory replay and reflection, and thoughts about the future—could be generated by such a self-maintaining, predictive encoding system.

Due to the developed event, event boundary, event schema, and episode encodings, these imaginations typically would not come in the form of very concrete sensory or motor images, but they could also be established on more abstract, conceptual levels. A mental image of a particular concept—such as a “ball,” a “surface,” a “container,” or even a “democracy”—would be encoded by a distributed but consistent set of active predictive encodings, which predict the believed characteristics of the particular concept. The consistency of the set would be essentially akin to a distributed neural attractor, which approximates a temporary free-energy minimum. In other words, the active predictive encodings, which predict activities of each other, are in agreement, such that none of the active encodings significantly contradict others. Note how the agreement is closely related to the error residual after activity adaptation, as it was quantified in a simplified manner in Equation (3). The remaining residual after activity adaptation essentially characterizes the (believed) uncertainty about the concept. A concept composition corresponds to an attractor that integrates several active concepts.

For example, the mental image of a ball may be constituted by top-down predictive encodings of roundness features and a full circular shape as well as imprecise weight estimates, for example, in the form of predicted tactile and proprioceptive feedback upon bodily interactions. Additionally, spatial predictive encodings may yield size and volume estimates, as well as possibly an imprecise default location—as in “in front of the eyes.” Temporal predictive encodings may encode typical ball behavior, such as rolling and bouncing, as well as the forces that typically interact with a ball, such as forces caused by hands, feet, head, other body parts, tools, and other items. Imaginable interactions essentially relate these other concepts to the ball, thus enabling the imagining of particular scenarios, such as, for example, a soccer stadium, a penalty kick, or a goal—or in tennis a serve, an ace, or a return. Figure 2A illustratively shows a distributed predictive encoding network, which characterizes some of the semantics of a ball.

In a similar manner, the concept of a “bowl” can be illustrated. Figure 2B shows some of the involved predictive encodings. Shown are a top-down encoding that predicts how the bowl may look and a relative spatial encoding, which predicts where the bowl may be situated. Moreover, two temporal encodings specify how another entity may be affected when coming in contact with the bowl and how the bowl may behave, when a force affects it Note how the bowl is much more stable than the ball, which is encoded in the temporal predictive encodings of possible motion dynamics. Note also how the bowl specifies an attracting subspace, characterizing its hollow area, which can be interpreted as a characterization of a “container” concept.

Concept compositions then may combine several concepts into an integrative attractor. Figure 2C illustratively shows how the concept composition of “a ball lies in a bowl” may be encoded in a distributed attractor that consists of a set of active predictive encodings. The ball and bowl concepts are both active and temporarily related to each other in a relative spatial frame of reference. The illustrated relative spatial encodings predict that the ball is most likely somewhat smaller than the bowl and that the ball is situated somewhere within the hollow area of the bowl. Temporal predictive encoding activities may adapt due to the activated spatial relationship, seeking an agreeable free energy minimum. For example, the temporal predictive encodings about interaction consequences may cancel each other out, such that the ball may be imagined to lie stably within the hollow area of the bowl. Moreover, the predictions about possible motion dynamics may be adapted such that sudden motion onsets—particularly of the ball—become less likely, unless the bowl is moved.

As a result, the concept composition may predict that the ball is unlikely to roll out of the bowl, that it probably lies stably inside, and that it probably remains inside even when the bowl is moved. Moreover, the composite encoding is even able to generate a visual image, where a prototypical ball (such as the soccer ball shown) lies within the bowl. Note also how the words of the sentence may be mapped onto the concepts and how they are constraining the concept composition. The verb “lies” implies stability, such that the estimates of the ball rolling should be low as should estimates about the bowl currently moving. Moreover, the preposition “in” implies that the subject (the “ball”) can be found in the object (the “bowl”), which is only possible if an inside area can be defined and is accessible. As a consequence, the reversed sentence: “a bowl lies in a ball” is much harder to imagine because a ball does not have an accessible interior, as also pictured in the “ball” concept illustration (Figure 2A).

## 5. Summary and outlook

This paper has proposed a path toward a unifying sub-symbolic computational theory of cognition. The proposal suggests that thought—including thoughts about possibly hypothetical, highly abstracted, imagined scenarios, and behavior in such scenarios—is generated by sets of currently active encodings. The encodings structure themselves based on the gathered sensory-motor experiences, are predictive in nature, and comprise top-down, spatial, and temporal predictive components. Event-oriented abstractions enable the learning of event schemata and integrative episodic encodings. A particular concept about our world is encoded in the form of an approximately consistent set of active predictive encodings.

Active inference drives behavior in an epistemic and goal-directed manner, with the aim to maintain internal system homeostasis. Similarly, attention is driven by active inference, causing the activation of consistent sets of predictive encodings and the consideration of possible temporal progressions through these encodings. As a result, sets of predictive encodings essentially encode the perception or imagining of a scenario and the potential changes in this scenario over time. The main propositions of the proposed unifying theory are summarized in Table 2. Note that these propositions are certainly not all new, but their integrative composition is.

**Table 2:** **Main propositions toward a unifying, sub-symbolic, computational theory of cognition**.

1.	The brain is a modular, probabilistic, predictive encoding system that continuously strives to minimize free energy in a distributed manner;
2.	Predictive encodings are separated into temporal, spatial, and top-down predictive encodings;
3.	Modularity develops in the brain to be able to flexibly relate particular predictive encodings across space and time and to be able to form effective abstractions and generalizations;
4.	Behavior, attention, and thought are anticipatory because they are generated by active inference mechanisms, which activate temporal predictive encodings inversely due to differences in current and desired internal homeostatic states, which in turn activate associated forces, motor behavior, attention, and thought itself;
5.	Concepts are approximately consistent free energy minima in a distributed set of active, interconnected predictive encodings;
6.	Concept compositions are combinations of such concepts that are temporarily consistently related to each other (approximating a larger, more distributed free energy minimum);
7.	Particular scenarios, such as the current or an imagined world state, are perceived, imagined, or remembered in the form of compositional concepts;
8.	Episodes are perceived, imagined, or remembered in the form of compositional concepts and a concatenation of event schema-encodings (typically on multiple levels of abstraction), which specify how the scenario changes (or may change or changed) over time;
9.	The cognitive pursuance of a particular “idea” or a particular “thought” corresponds to the active exploration of concepts, concept compositions, scenarios, and/or episodes by means of event schema-based activity changes over time.

Clearly, many challenges remain for developing an actual implementation of the theory, filling in details, and verifying (or falsifying) its propositions and predictions. Various laboratories are working toward developing aspects of the herein proposed theory unification, but clearly a fully integrative implementation is missing. Verifications of the resulting system abilities are pending as well at this point. Nonetheless, the propositions put forward in Table 2 can be verified, falsified, or further differentiated. The predictions of the sketched-out unified theory about how concepts and compositional concepts are encoded and how they develop, for example, can be questioned and falsified. Also, the involved learning mechanisms can be further investigated.

It should also be emphasized that a couple of important aspects have not been addressed by the proposed unification. These aspects particularly include social and language dimensions. While self-motivated, goal-directed behavior has been considered, the concept of intentionality has not been addressed. This is because social aspects were neglected, that is, I have not addressed how the system may encode other agents in the environment. While other agents may generally be perceived as items, clearly our brain encodes other animals and particularly humans differently from inanimate items in the environment (Amodio and Frith, 2006; Chouchourelou et al., 2013). Predictive encoding capabilities establish themselves in prefrontal cortical areas, which seem to allow (i) the separation and integration of predictive self-representations (Butz, 2008) from representations of others in the social context and (ii) the attribution of intentions and individualized knowledge to others (Frith and Frith, 2005; Amodio and Frith, 2006). Albeit I believe that similar predictive encoding concepts can establish such forms of encodings, I leave further elaborations on this point for future research.

The language dimension stands in close relation to the social dimension, as language without the drive for social communication hardly makes any sense. The fact that language shapes the way we think is at this point rather clearly established (e.g., Griffin and Bock, 2000; Thibodeau and Boroditsky, 2013). However, also many researchers nowadays suggest that thought comes before language and enables language learning in the first place (e.g., Mandler, 2004; Evans, 2015). The proposed unified theory essentially puts forward how thought may be structured before language, grounding thought encodings in sensorimotor experiences and abstractions thereof. Propositions that such structures make a system language ready have been put forward (e.g., Pulvermüller and Fadiga, 2010; Pastra and Aloimonos, 2012), but require further elaboration.

Before adding language, though, implementations of the sketched-out pathway toward conceptualized thought are necessary, enabling proofs of the outlined principles, including the progression toward abstract predictive encodings in their various forms. How may such an implementation be accomplished? One important challenge in this respect is the generation of data, that is, a large amount of actual, self-motivatedly generated bodily experiences, which are necessary to simulate cognitive development. The capabilities of the currently available robots are clearly too limited in this respect, allowing the active gathering of data only over a couple of days at best. A way out of this dilemma seems to be the use of reasonable realistic simulators of virtual realities. Interestingly, the developments pushed forward by the computer games industry may be helpful in this respect. Game engines, such as Unity3D (https://unity3d.com/), the CryEngine (http://cryengine.com/), or the Unreal Engine (https://www.unrealengine.com/), offer themselves as sufficiently realistic environments in which an artificial agent can gather large amounts of data even faster than real time. Also research driven VR engines may be suitable, such as the MuJoCo advanced physics simulation (http://www.mujoco.org/). The recent impact of a deep learning artificial neural architecture in playing some of the traditional Atari arcade games human-competitively points out that such simulations are possible (Mnih et al., 2015). Note that in this particular case, however, no generative system was employed and no real “understanding” of the games by the system was shown.

As long as the artificial self-developing system implementation, which is based on the proposed unifying theory, is equipped with (i) suitable sensory and motor capabilities and (ii) a suitably structured internal system of motivations, it may be released into any available, sufficiently rich virtual reality simulation. Analyses of the actual cognitive development that can be accomplished by such self-developing, artificial, cognitive creatures will be highly revealing. The resulting, potential insights may shed further light on (i) the fundamental functional and computational principles implemented by our brains, (ii) how cognitive development actually proceeds mechanistically, and (iii) how cognition itself unfolds in sensorimotor experience-grounded, predictive encodings.

## Author contributions

The author confirms being the sole contributor of this work and approved it for publication.

### Conflict of interest statement

The author declares that the research was conducted in the absence of any commercial or financial relationships that could be construed as a potential conflict of interest. The reviewer MZ and handling Editor declared their shared affiliation, and the handling Editor states that the process nevertheless met the standards of a fair and objective review.

## Footnotes

^1^Note that I intentionally do not delve deeper into distinctions between body image, body schema, kinematic body models, postural schema, peripersonal space, etc. here.
